# Supplementary material for: Methicillin-resistant and methicillin-sensitive Staphylococcus aureus isolates from skin and nares of Brazilian children with atopic dermatitis demonstrate high level of clonal diversity
Source: PLoS One. 2022 Nov 3;17(11):e0276960. doi: 10.1371/journal.pone.0276960 (PMC9632840; doi:10.1371/journal.pone.0276960)
Supplement: S1 Table — (DOC) [file pone.0276960.s001.doc]

**Table S1. Description of the PCR conditions used to confirm staphylococcal identification**

| **Species** | **Oligonucleotide**  **Sequence (5’- 3’)** | **Concentration**  **(μM)** | **Amplicon size (pb)** | **PCR cycle** | **Reference** |
| --- | --- | --- | --- | --- | --- |
| *Staphylococcus aureus* | SA442-1- AATCTTTGTCGGTACACG  ATATTCTTCACG  SA442-2- CGTAATGAGATTTCAGTA  GATAATACAACA | 0.4 | 108 | 94 ºC/1 min  55 ºC/1 min  72 ºC/1 min  (30 X) | [12] |
| *Staphylococcus epidermidis* | SEpF- CAGTTAATCGGTATGAGAGC  SEpR- CTGTAGAGTGACAGTTTGGT | 0.4 | 219 | 94 ºC/1 min  55 ºC/1 min  72 ºC/1 min  (30 X) | [13] |
| *Staphylococcus haemolyticus* | SH1- GGTCGCTTAGTCGGAACAAT  SH2- CACGAGCAATCTCATCACCT | 0.5 | 286 | 94 ºC/1 min  55 ºC/1 min  72 ºC/1 min  (30 X) | [14] |
| *Staphylococcus hominis* | homF- TACAGGGCCATTTAAAGACG  homR- GTTTCTGGTGTATCAACACC | 0.2 | 177 | 95 ºC/30 s  58 ºC/30 s  72 ºC/70 s  (30 X) | [15] |
| *Staphylococcus saprophyticus* | hrcAF- GACCTTTCCTCTACATTGAG  hrcA- CCTGATGTAAACACAACCAC | 0.25 | 164 | 94 ºC/45 s  56 ºC/30 s  72 ºC/30 s  (30 X) | [16] |
