# Supplementary material for: Methicillin-resistant and methicillin-sensitive Staphylococcus aureus isolates from skin and nares of Brazilian children with atopic dermatitis demonstrate high level of clonal diversity
Source: PLoS One. 2022 Nov 3;17(11):e0276960. doi: 10.1371/journal.pone.0276960 (PMC9632840; doi:10.1371/journal.pone.0276960)
Supplement: S2 Table — Light grey- Absent; Dark grey- Present; SDA- Child without atopic dermatitis; SCORAD- Scoring atopic dermatitis; SCORAD 1- Mild; SCORAD 2- Moderate; SCORAD 3- Severe; na- Not applicable; F- Female; M- Male; Sa- S. aureus; Sc- S. capitis; Se- S. epidermidis; Sh- S. haemolyticus; Sn- S. hominis; Ss- S. saprophyticus; O(s)- Another coagulase-negative Staphylococcus spp.; MRSA- Methicillin-resistant S. aureus; Sa PVL+- S. aureus presenting the Panton-Valentine leukocidin genes; MR-CoNS- Methicillin-resistant coagulase-negative Staphylococcus; Child 27*- Child with atopic dermatitis without a swab from non-lesional skin. (DOC) [file pone.0276960.s002.doc]

**Table S2. General characteristics of the 42 children included in the study**

| **AD Child** | **SCORAD** | **Gender** | **Age**  **(years)** | **Colonized by:** | | | | | | | | | | | | | | | | | | | | | | | |
| --- | --- | --- | --- | --- | --- | --- | --- | --- | --- | --- | --- | --- | --- | --- | --- | --- | --- | --- | --- | --- | --- | --- | --- | --- | --- | --- | --- |
|  |  |  |  | **Nares** | | | | | | | **Lesional skin** | | | | | | | **Non-lesional skin** | | | | | | | **MRSA** | **Sa**  **PVL+** | **MR-**  **CoNS** |
|  |  |  |  | **Sa** | **Sc** | **Se** | **Sh** | **Sn** | **SS** | **O(s)** | **Sa** | **Sc** | **Se** | **Sh** | **Sn** | **Ss** | **O(s)** | **Sa** | **Sc** | **Se** | **Sh** | **Sn** | **Ss** | **O(s)** |  |  |  |
| 1 | 2 | M | 8 | 1 | 0 | 1 | 0 | 0 | 0 | 0 | 1 | 0 | 1 | 0 | 0 | 0 | 0 | 1 | 0 | 1 | 0 | 0 | 0 | 0 | 1 | 0 | 1 |
| 2 | 3 | F | 8 | 1 | 0 | 0 | 0 | 0 | 0 | 0 | 1 | 0 | 0 | 0 | 0 | 0 | 0 | 1 | 0 | 1 | 0 | 0 | 0 | 0 | 1 | 1 | 1 |
| 3 | 2 | F | 9 | 1 | 0 | 1 | 0 | 0 | 0 | 0 | 1 | 0 | 1 | 0 | 1 | 1 | 0 | 1 | 0 | 1 | 1 | 0 | 1 | 0 | 0 | 0 | 1 |
| 4 | 3 | F | 6 | 1 | 0 | 1 | 0 | 0 | 0 | 0 | 1 | 0 | 1 | 0 | 0 | 0 | 1 | 0 | 0 | 1 | 0 | 0 | 0 | 1 | 0 | 0 | 1 |
| 5 | 1 | M | 7 | 1 | 0 | 1 | 0 | 0 | 0 | 0 | 1 | 1 | 1 | 0 | 0 | 0 | 0 | 1 | 0 | 1 | 0 | 1 | 0 | 0 | 0 | 0 | 1 |
| 6 | 2 | F | 7 | 1 | 0 | 1 | 0 | 0 | 0 | 1 | 1 | 0 | 0 | 0 | 0 | 0 | 0 | 1 | 0 | 0 | 0 | 0 | 0 | 0 | 0 | 0 | 1 |
| 7 | 2 | F | 5 | 1 | 0 | 1 | 0 | 0 | 0 | 0 | 1 | 1 | 1 | 0 | 0 | 0 | 0 | 1 | 1 | 1 | 0 | 0 | 0 | 0 | 0 | 0 | 1 |
| 8 | 2 | M | 9 | 1 | 0 | 0 | 0 | 0 | 0 | 0 | 1 | 0 | 0 | 0 | 0 | 0 | 0 | 0 | 0 | 1 | 0 | 1 | 1 | 0 | 1 | 0 | 1 |
| 9 | 2 | F | 5 | 1 | 0 | 0 | 0 | 0 | 0 | 0 | 1 | 0 | 0 | 0 | 0 | 0 | 0 | 1 | 0 | 1 | 0 | 1 | 0 | 0 | 0 | 0 | 1 |
| 10 | 2 | M | 7 | 1 | 0 | 1 | 0 | 0 | 0 | 0 | 1 | 0 | 0 | 0 | 0 | 0 | 0 | 1 | 0 | 1 | 1 | 1 | 0 | 0 | 0 | 1 | 1 |
| 11 | 2 | M | 3 | 1 | 0 | 1 | 0 | 0 | 0 | 0 | 1 | 0 | 0 | 0 | 0 | 0 | 0 | 0 | 0 | 1 | 0 | 0 | 0 | 0 | 0 | 1 | 1 |
| 12 | 2 | F | 5 | 1 | 0 | 1 | 0 | 0 | 0 | 0 | 1 | 0 | 1 | 0 | 1 | 0 | 0 | 1 | 0 | 1 | 0 | 1 | 0 | 0 | 1 | 0 | 1 |
| 13 | 2 | F | 6 | 1 | 0 | 0 | 0 | 0 | 0 | 0 | 1 | 0 | 1 | 1 | 0 | 0 | 0 | 1 | 1 | 0 | 0 | 0 | 0 | 0 | 0 | 0 | 1 |
| 14 | 1 | M | 9 | 1 | 1 | 0 | 0 | 0 | 0 | 0 | 1 | 0 | 1 | 0 | 0 | 0 | 1 | 1 | 1 | 1 | 1 | 1 | 0 | 1 | 0 | 1 | 1 |
| 15 | 2 | M | 7 | 1 | 0 | 1 | 0 | 0 | 0 | 0 | 1 | 0 | 1 | 0 | 0 | 0 | 0 | 1 | 0 | 1 | 0 | 0 | 0 | 0 | 0 | 1 | 1 |
| 16 | 2 | F | 6 | 1 | 0 | 0 | 0 | 0 | 0 | 0 | 1 | 0 | 1 | 0 | 0 | 0 | 0 | 0 | 0 | 1 | 0 | 1 | 0 | 1 | 0 | 0 | 1 |
| 17 | 2 | M | 5 | 1 | 0 | 0 | 0 | 0 | 0 | 0 | 1 | 0 | 1 | 0 | 1 | 0 | 0 | 1 | 0 | 1 | 0 | 0 | 0 | 0 | 1 | 1 | 1 |
| 18 | 1 | F | 2 | 1 | 0 | 1 | 0 | 1 | 0 | 0 | 1 | 0 | 1 | 0 | 1 | 0 | 0 | 1 | 0 | 1 | 0 | 1 | 1 | 0 | 1 | 1 | 1 |
| 19 | 2 | F | 6 | 1 | 0 | 1 | 0 | 0 | 0 | 0 | 1 | 0 | 1 | 0 | 0 | 0 | 0 | 1 | 0 | 1 | 0 | 1 | 0 | 1 | 1 | 0 | 1 |
| 20 | 2 | F | 4 | 1 | 0 | 0 | 0 | 0 | 0 | 0 | 1 | 0 | 0 | 0 | 0 | 0 | 0 | 1 | 0 | 0 | 0 | 0 | 0 | 0 | 0 | 1 | 0 |
| 21 | 2 | F | 8 | 1 | 0 | 1 | 0 | 0 | 0 | 0 | 1 | 1 | 1 | 0 | 0 | 0 | 0 | 1 | 1 | 1 | 1 | 0 | 1 | 0 | 0 | 0 | 1 |
| 22 | 1 | F | 6 | 1 | 0 | 1 | 0 | 0 | 0 | 0 | 1 | 0 | 1 | 0 | 0 | 0 | 0 | 0 | 1 | 1 | 0 | 1 | 0 | 0 | 0 | 0 | 1 |
| 23 | 2 | F | 9 | 1 | 1 | 1 | 0 | 0 | 0 | 0 | 1 | 0 | 1 | 0 | 1 | 0 | 0 | 1 | 0 | 1 | 0 | 0 | 0 | 0 | 1 | 0 | 1 |
| 24 | 1 | M | 9 | 1 | 0 | 0 | 0 | 0 | 0 | 1 | 1 | 0 | 1 | 0 | 0 | 0 | 0 | 1 | 0 | 1 | 1 | 1 | 0 | 1 | 1 | 0 | 1 |
| 25 | 2 | F | 3 | 1 | 0 | 1 | 0 | 1 | 0 | 0 | 1 | 0 | 1 | 0 | 1 | 0 | 0 | 1 | 0 | 1 | 0 | 1 | 0 | 0 | 0 | 0 | 1 |
| 26 | 2 | F | 2 | 1 | 0 | 1 | 0 | 0 | 0 | 0 | 1 | 0 | 1 | 0 | 0 | 1 | 0 | 1 | 1 | 1 | 0 | 0 | 1 | 0 | 0 | 1 | 1 |
| 27* | 3 | F | 10 | 1 | 0 | 0 | 0 | 0 | 0 | 0 | 1 | 0 | 0 | 0 | 0 | 0 | 0 | na | na | na | na | na | na | na | 0 | 0 | 0 |
| 28 | 3 | M | 3 | 1 | 0 | 1 | 0 | 0 | 0 | 0 | 1 | 0 | 0 | 0 | 0 | 0 | 0 | 1 | 0 | 1 | 0 | 0 | 1 | 0 | 0 | 0 | 1 |
| 29 | 1 | M | 7 | 0 | 0 | 0 | 0 | 0 | 0 | 1 | 1 | 0 | 0 | 0 | 0 | 0 | 0 | 1 | 0 | 1 | 0 | 1 | 0 | 0 | 0 | 0 | 1 |
| 30 | 2 | F | 6 | 1 | 0 | 0 | 0 | 0 | 0 | 1 | 1 | 0 | 0 | 0 | 0 | 0 | 0 | 1 | 0 | 0 | 0 | 0 | 0 | 0 | 0 | 0 | 0 |

| **Non-AD Child** | **SCORAD** | **Gender** | **Age**  **(years)** | **Colonized by:** | | | | | | | | | | | | | | | | | | | | | | | |
| --- | --- | --- | --- | --- | --- | --- | --- | --- | --- | --- | --- | --- | --- | --- | --- | --- | --- | --- | --- | --- | --- | --- | --- | --- | --- | --- | --- |
|  |  |  |  | **Nares** | | | | | | | **Lesional skin** | | | | | | | **Non-lesional skin** | | | | | | | **MRSA** | **Sa**  **PVL+** | **MR-**  **CoNS** |
|  |  |  |  | **Sa** | **Sc** | **Se** | **Sh** | **Sn** | **Ss** | **O(s)** | **Sa** | **Sc** | **Se** | **Sh** | **Sn** | **Ss** | **O(s)** | **Sa** | **Sc** | **Se** | **Sh** | **Sn** | **Ss** | **O(s)** |  |  |  |
| SDA1 | na | M | 6 | 1 | 0 | 1 | 0 | 1 | 0 | 0 | na | na | na | na | na | na | na | 1 | 0 | 1 | 0 | 1 | 0 | 0 | 0 | 0 | 1 |
| SDA2 | na | F | 2 | 0 | 0 | 1 | 0 | 1 | 0 | 0 | na | na | na | na | na | na | na | 1 | 0 | 1 | 0 | 1 | 0 | 0 | 0 | 0 | 1 |
| SDA3 | na | M | 5 | 1 | 0 | 1 | 0 | 0 | 0 | 0 | na | na | na | na | na | na | na | 0 | 0 | 0 | 0 | 1 | 0 | 0 | 0 | 0 | 1 |
| SDA4 | na | F | 2 | 1 | 1 | 1 | 0 | 0 | 0 | 1 | na | na | na | na | na | na | na | 0 | 0 | 1 | 0 | 1 | 0 | 1 | 0 | 0 | 1 |
| SDA5 | na | F | 8 | 0 | 0 | 1 | 0 | 0 | 1 | 0 | na | na | na | na | na | na | na | 0 | 0 | 1 | 1 | 0 | 1 | 0 | 0 | 0 | 1 |
| SDA6 | na | F | 2 | 1 | 1 | 0 | 0 | 1 | 1 | 0 | na | na | na | na | na | na | na | 1 | 0 | 0 | 0 | 1 | 1 | 0 | 1 | 1 | 1 |
| SDA7 | na | M | 4 | 0 | 0 | 1 | 1 | 1 | 0 | 0 | na | na | na | na | na | na | na | 0 | 0 | 0 | 1 | 1 | 0 | 0 | 0 | 0 | 1 |
| SDA8 | na | M | 2 | 0 | 0 | 1 | 1 | 1 | 1 | 0 | na | na | na | na | na | na | na | 0 | 1 | 1 | 1 | 1 | 1 | 0 | 0 | 0 | 1 |
| SDA9 | na | F | 2 | 0 | 0 | 1 | 0 | 1 | 0 | 0 | na | na | na | na | na | na | na | 0 | 0 | 1 | 0 | 1 | 0 | 1 | 0 | 0 | 1 |
| SDA10 | na | F | 10 | 1 | 0 | 1 | 0 | 0 | 0 | 0 | na | na | na | na | na | na | na | 0 | 1 | 1 | 0 | 1 | 1 | 0 | 0 | 0 | 1 |
| SDA11 | na | M | 6 | 1 | 0 | 1 | 0 | 0 | 0 | 1 | na | na | na | na | na | na | na | 1 | 0 | 1 | 0 | 1 | 0 | 0 | 0 | 1 | 1 |
| SDA12 | na | M | 8 | 1 | 0 | 1 | 0 | 0 | 1 | 0 | na | na | na | na | na | na | na | 1 | 0 | 0 | 0 | 1 | 0 | 0 | 0 | 1 | 0 |


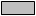
- Absent;
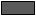
- Present; SDA- Child without atopic dermatitis; SCORAD- *Scoring atopic dermatitis*; SCORAD 1- Mild; SCORAD 2- Moderate; SCORAD 3- Severe; na- Not applicable; F- Female; M- Male; Sa- *S. aureus;* Sc- *S. capitis;* Se- *S. epidermidis;* Sh- *S. haemolyticus;* Sn- *S. hominis;* Ss- *S. saprophyticus;* O(s)- Another coagulase-negative *Staphylococcus* spp.; MRSA- Methicillin-resistant *S. aureus;* Sa PVL+- *S. aureus* presenting the Panton-Valentine leukocidin genes; MR-CoNS- Methicillin-resistant coagulase-negative *Staphylococcus;* Child 27*- Child with atopic dermatitis without a swab from non-lesional skin.
