# Supplementary material for: Methicillin-resistant and methicillin-sensitive Staphylococcus aureus isolates from skin and nares of Brazilian children with atopic dermatitis demonstrate high level of clonal diversity
Source: PLoS One. 2022 Nov 3;17(11):e0276960. doi: 10.1371/journal.pone.0276960 (PMC9632840; doi:10.1371/journal.pone.0276960)
Supplement: Attachment — Submitted filename: Response to Reviewers.doc [file pone.0276960.s003.doc]

**To**

**Feroze Kaliyadan, M.D.**

**Academic Editor in PLOS ONE**

**Ref. No.: PONE-D-22-24445**

Dear Editor,

I am submitting the revised version of the manuscript Ref. No.: PONE-D-22-24445.

We have evaluated the journal requirements and the comments of reviewers. All the responses were answered in a point-by-point discussion. The manuscript was revised to improve English.

Kind Regards,

Kátia R. N. dos Santos

**Journal Requirements:**

When submitting your revision, we need you to address these additional requirements.

1. Please ensure that your manuscript meets PLOS ONE's style requirements, including those for file naming. The PLOS ONE style templates can be found at 
<https://journals.plos.org/plosone/s/file?id=wjVg/PLOSOne_formatting_sample_main_body.pdf> and 
<https://journals.plos.org/plosone/s/file?id=ba62/PLOSOne_formatting_sample_title_authors_affiliations.pdf>

**Done.**

**“Author contributions” section removed.**

**Tables 1 and 2 formatted according to the style requirements: text center-aligned; font size changed; extra spaces removed.**

**Position of the legends of all figures formatted.**

**Position of the Table 1 footnote formatted.**

2. We note that you have included the phrase “data not shown” in your manuscript. Unfortunately, this does not meet our data sharing requirements. PLOS does not permit references to inaccessible data. We require that authors provide all relevant data within the paper, Supporting Information files, or in an acceptable, public repository. Please add a citation to support this phrase or upload the data that corresponds with these findings to a stable repository (such as Figshare or Dryad) and provide and URLs, DOIs, or accession numbers that may be used to access these data. Or, if the data are not a core part of the research being presented in your study, we ask that you remove the phrase that refers to these data.

**We removed the phrase “data not shown” (line 216).**

3. Please review your reference list to ensure that it is complete and correct. If you have cited papers that have been retracted, please include the rationale for doing so in the manuscript text, or remove these references and replace them with relevant current references. Any changes to the reference list should be mentioned in the rebuttal letter that accompanies your revised manuscript. If you need to cite a retracted article, indicate the article’s retracted status in the References list and also include a citation and full reference for the retraction notice.

**We changed reference 27 for another study as the previously one presented an erratum.**

**The reference 21 also presents an erratum. However, we did not change this reference since it is largely used for SCC*mec* typing and it has 189 citations according to Pubmed.**

**Review Comments to the Author**

**Reviewer #1:**

1. Can the title be rephrased as: Methicillin-resistant and methicillin sensitive Staphylococcus aureus isolates from skin and nares of Brazilian children with atopic dermatitis demonstrate high level of clonal diversity. This would make it more appealing and interesting.

**Done.**

2. Highlight in the introduction part: What is the significance of the study? What is the significance and relevance of molecular characterization of MRSA in AD?

**We added two sentences in the last paragraph of the introduction answering those questions (lines 82 and 83; 87-90).**

3. The paper needs grammar check. A few grammatical errors are found in the paper.
e.g.: page 3, line 60-61
"The presence of strains carrying genes for toxins is reported, which may be associated with triggering, maintaining, and increasing of the inflammatory process".
This sentence could be framed as below.
"The presence of virulence and toxin genes has been reported among staphylococcal isolates from AD, which may be associated with triggering, sustaining, and amplifying the inflammatory process".
The authors are advised to have the paper checked by a native English speaker.

**Done.**

4. Any mention of abbreviation should be with the full term for the first time and the abbreviations written in the parenthesis. Subsequently only the abbreviation can be used thereof. This has not been followed.
e.g.:
a. page 3, line 65. MRSA is mentioned in parenthesis for community methicillin-resistant
b. page 4, line 88. IPPMG
**Done.**

5. There are some instances of incomplete sentences.
e.g.: page 5, line 104-105.
"Unable to collect a swab from the non-lesional skin of one AD child".
This could be written as, " We were unable to collect a swab from the non-lesional skin of one AD child.

**Done.**

**Reviewer #2:**

Well conducted study. The findings, especially regarding the high prevalence of MR-CoNS of different species colonizing the skin and nares of AD patients are valuable.
Have the following clarifications/ comments:
-Were there any frank infections like cellulitis/ abscesses/ impetigo noted in the study patients.

**As stated in the lines 102 and 103, patients with any sign of infection were not included in the study.**

-Severity scoring (clinical severity) would perhaps have added more value

**The severity of the disease was assessed in the day of the sample collection using the SCORAD index (lines 100 and 101). We presented the distribution of the patients according to the SCORAD in lines 167 and 168. We also mention that some patients with moderate and severe AD only had *S. aureus* recovered from skin sites (lines 211-214; 266-268; 301-305).**

-In the children without AD with Staph aureus colonisation, were there any risk factors present for acquiring the same.

**The children without AD were siblings of AD children (lines 98-100), which could be a risk factor for acquiring *S. aureus*, since these children have close contact with AD patients (lines 306-315).**
